# A theory-based intervention to promote medication adherence in patients with rheumatoid arthritis: A randomized controlled trial

**DOI:** 10.1007/s10067-020-05224-y

**Published:** 2020-06-25

**Authors:** Shahnaz Asgari, Mahnaz Abbasi, Kyra Hamilton, Yu-Pin Chen, Mark D. Griffiths, Chung-Ying Lin, Amir H. Pakpour

**Affiliations:** 1 Student Research Committee, Qazvin University of Medical Sciences, Qazvin, Iran; 2 Metabolic Disease Research Center, Research Institute for Prevention of Non-communicable Diseases, Qazvin University of Medical Sciences, Qazvin, Iran; 3 School of Applied Psychology, Menzies Health Institute Queensland, Griffith University, Brisbane, Queensland Australia; 4 Department of Orthopedic Surgery, Wan Fang Hospital, School of Medicine, College of Medicine, Taipei Medical University, Taipei, Taiwan; 5 Psychology Department, Nottingham Trent University, Nottingham, UK; 6 Department of Rehabilitation Sciences, Faculty of Health & Social Sciences, The Hong Kong Polytechnic University, Hung Hom, Hong Kong; 7 Social Determinants of Health Research Center, Research Institute for Prevention of Non-communicable Diseases, Qazvin University of Medical Sciences, Shahid Bahounar BLV, Qazvin, 3419759811 Iran; 8 Department of Nursing, School of Health and Welfare, Jönköping University, Jönköping, Sweden

**Keywords:** Health action process approach, Medicine adherence, Rheumatoid arthritis, Social cognition

## Abstract

**Introduction/objectives:**

Adherence to prescribed medication regimens is fundamental to the improvement and maintenance of the health of patients with rheumatoid arthritis. It is therefore important that interventions are developed to address this important health behavior issue. The aim of the present study was to design and evaluate a theory-based intervention to improve the medication adherence (primary outcome) among rheumatoid arthritis patients.

**Methods:**

The study adopted a pre-registered randomized controlled trial design. Rheumatoid arthritis patients were recruited from two University teaching hospitals in Qazvin, Iran from June 2018 to May 2019 and randomly assigned to either an intervention group (*n* = 100) or a treatment-as-usual group (n = 100). The intervention group received a theory-based intervention designed based on the theoretical underpinnings of the health action process approach (HAPA). More specifically, action planning (making detailed plans to follow medication regimen), coping planning (constructing plans to overcome potential obstacles that may arise in medication adherence), and self-monitoring (using a calendar to record medication adherence) of the HAPA has been used for the treatment. The treatment-as-usual group received standard care.

**Results:**

Data analysis was conducted based on the principle of intention to treat. Using a linear mixed-effects model (adjusted for age, sex, medication prescribed, and body mass index), the results showed improved medication adherence scores in the intervention group (loss to follow-up = 16) compared to the treatment-as-usual group (loss to follow-up = 12) at the 3-month (coefficient = 3.9; SE = 0.8) and 6-month (coefficient = 4.5; SE = 0.8) follow-up. Intervention effects on medication adherence scores were found to be mediated by some of the theory-based HAPA variables that guided the study.

**Conclusion:**

The results of the present study support the use of a theory-based intervention for improving medication adherence among rheumatoid arthritis patients, a group at-risk of not adhering to medication regimens.

**Trial registration (in Iranian Registry of Clinical Trials):**

irct.ir, IRCT20180108038271N1

**Table:** 

**Key Points** *• Theoretical underpinnings of the health action process approach are useful to improve medication adherence for RA patients.*

**Electronic supplementary material:**

The online version of this article (10.1007/s10067-020-05224-y) contains supplementary material, which is available to authorized users.

## Introduction

Rheumatoid arthritis (RA) is a chronic autoimmune disorder that affects synovial joints, resulting in severe disability and morbidity [1]. With disease progression, RA can also present with extra-articular manifestations such as rheumatoid nodules, pulmonary involvement or vasculitis, and systemic comorbidities [2]. RA affects approximately 0.5 to 1% of the population worldwide [3], with reported prevalence of 0.33% in Iran [4]. Further, RA has been ranked as the 42nd highest contributor to global disability of 291 conditions studied, carrying a substantial burden for both the individual and society more generally [5]. Although modern medicine has advanced treatments for the effective management of RA, the long-term medical, social, and economic consequences are still underestimated [6].

Drug therapy for RA patients is especially important to improve radiographic disease progression, physical function, and quality of life [7–9]. Although the drug treatment for RA patients is complex (e.g., measurement on adherence is difficult to establish and outcomes are dependent on a range of variables), prior research has demonstrated an association between higher medication adherence and better clinical response to therapies in RA patients [10, 11]. Thus, improving medication adherence in RA patients may, in turn, help to improve their health outcomes. Despite this, a recent meta-analysis revealed that only 66% of all patients are actually adherent to their RA treatment regimen [12]. Strategies to enhance medication adherence among RA patients are therefore of value to help maximize the efficacy of treatment and minimize the course of RA progression.

Many researchers and clinicians have observed the problem of medication nonadherence among RA patients. Consequently, programs for improving medication adherence have been developed and studied [13–16]. However, the commonly used method of patient education in improving mediation adherence is questionable due to the reported limited short-term benefits and inconsistent effects [17]. This suggests that the use of education to improve medication adherence for patients with RA, although perhaps important, is not sufficient to change behavior to adhere to their treatment regimens. Other potential methods, such as electronic drug monitoring feedback [18] and theory-based intervention [19], are needed to examine their effectiveness in improving medication adherence for patients with RA.

Mechanisms proposed by a theory can help understand medication adherence more generally. Theory-based programs are demonstrated to be more effective in promoting behavior change compared with atheoretical campaigns [20–23]. However, although theory-based interventions are debated as necessary for the success of health programs [24], theory is often overlooked in intervention design and evaluation [25]. It is important that interventions using content based on theory provide an evaluation of the proposed mechanisms by which they are purported to affect behavior change [26]. The aim of the present study was to evaluate an intervention designed based on the theoretical underpinnings of the health action process approach (HAPA; for an overview of the HAPA and how it is used to change behavior see [27–29]) to improve adherence to medication regimens of patients with RA.

More specifically, important components in the self-regulatory phase of the HAPA (action planning, *how individuals make plans to perform their intended behavior*; coping planning, *how individuals make plans to overcome possible obstacles in undertaking their intended behavior*; and self-monitoring, *how individuals monitor their intended behavior*) [27–29] have been found to be effective in improving healthy behaviors (e.g., treatment adherence, eating fruits and vegetables, and sleep hygiene behaviors) among different populations [30–32]. For example, using action planning techniques, individuals can initiate a detailed plan to ensure they adhere to their medication regimen. Using coping planning techniques, individuals can overcome potential obstacles that may thwart their good intentions to adhere to their medication regimen. Using self-monitoring techniques, individuals can maintain medication adherence by regularly checking this behavior.

## Methods

The study adopted a pre-registered randomized controlled trial design: Iranian Registry of Clinical Trials (identifier umber: IRCT20180108038271N1; https://www.irct.ir/trial/28920). The ethics committee at Qazvin University of Medical Sciences (identification number: IR.QUMS.REC.1396.388) approved the study. All participants provided written informed consent prior to taking part in the study. More specifically, a preliminary session was held to deliver the study information. During the session, the first author was responsible for answering potential questions from the patients and consent forms were distributed for participants to read and sign.

### Participants and study design

The study adopted a single-blind randomized controlled trial (RCT) of 6-month duration and was conducted at two rheumatology outpatient clinics at two large university teaching hospitals in the city of Qazvin, Iran, from June 2018 to May 2019. Patients were eligible to participate if they had a confirmed diagnosis of RA according to the 2010 American College of Rheumatology (ACR)/European League Against Rheumatism (EULAR) criteria, were aged 18 years and older, and had the ability to understand written and spoken Persian language. Participants were excluded if they had a major psychiatric condition (e.g., schizophrenia, mood disorders, and substance-related disorders), severe kidney disease (this is because such patients need to have frequent consultations about their medication regime with their medical doctors which, in turn, might influence their medication adherence behaviors), the inability to take medication independently, and/or severe cognitive impairment (i.e., self-reported diagnosis of dementia and/or score < 24 on the Mini-Mental State Examination).

**Fig. 1:**
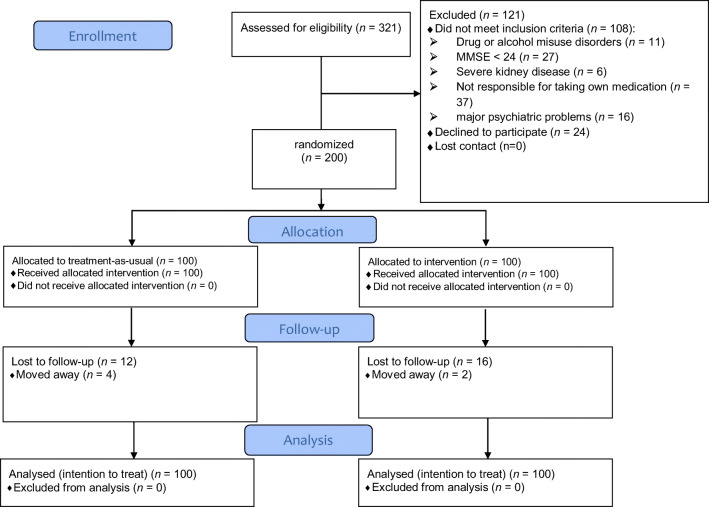
Flow diagram for random assignment of patients in the study

A flow diagram of the random assignment of patients in the study is presented in Fig. 1, and participant demographics are presented in Table 1. Recruiting research assistants were blinded to participant group allocation, and after baseline data were collected, participants were randomized to study conditions. The intervention contained three sessions, each spaced 1 week apart, and follow-up data were collected at 3 and 6 months post-intervention delivery. No additional tasks and resources were provided to the participants between sessions. Intervention sessions were conducted in a conference room in the outpatient setting while participants waited to attend their rheumatologist appointment. This waiting process usually takes about 1.5 h; thus, all consenting patients were able to participate in the sessions. As all patients needed to attend routine rheumatologist appointments on a regular basis, all three sessions were able to be completed. All participants completed the primary and secondary measures three times: baseline (before the intervention), 3 months after the intervention, and 6 months after the intervention.

**Table 1:** Baseline study sample characteristics and descriptive statistics for control variables

	Treatment-as-usual (*n* = 100)	Intervention (*n* = 100)	*p* value
Age, years; mean ± SD	55.0 ± 15.4	52.4 ± 13.6	0.24
Years of education; mean ± SD	7.1 ± 3.2	7.0 ± 3.0	0.52
Body mass index, kg/m^2^; mean ± SD	26.5 ± 5.2	25.9 ± 4.6	0.20
Marital status; *n* (%)			0.15
Single	12 (12.0)	9 (9.0)	
Married	67 (67.0)	58 (58.0)	
Divorced/widowed	21 (21.0)	33 (33.0)	
Sex; *n* (%)			0.66
Male	11 (11.0)	13 (13.0)	
Female	89 (89.0)	87 (87.0)	
Disease duration (years); mean ± SD	9.3 ± 6.5	8.9 ± 7.0	0.17
Anxiety; mean ± SD	8.2 ± 3.1	7.6 ± 3.2	0.15
Depression; mean ± SD	6.0 ± 2.2	6.1 ± 2.1	0.95
No. of DMARDs used; *n* (%)^a^			0.32
1 DMARD	10 (10.0)	8 (8.0)	
2 DMARDs	78 (78.0)	81 (81.0)	
3 ≥ DMARDs	12 (12.0)	11 (11.0)	

*SD*, standard deviation; *DMARD*, disease-modifying anti-rheumatic drugs

^a^Azathioprine, hydroxychloroquine, leflunomide, methotrexate, prednisone/prednisolone or sulfasalazine, adalimumab, methylprednisolone, etanercept, methotrexate, abatacept, infliximab, or rituximab

### Randomization and blinding

At baseline, research assistants, who were registered nurses, obtained signed written consent from each participant and administered questionnaires assessing the study’s primary and secondary outcomes (see sections of “Primary outcome of change in medication adherence”, “Secondary outcome measures of change in health outcome factors”, and “Secondary outcome measures of theory-based self-regulation factors” for details). Participants were then randomly assigned into one of two groups: an intervention group or a treatment-as-usual group. The randomization was conducted by a biostatistician at Tehran University of Medical Sciences who was not part of the study and who used a computer-generated block randomization (a combination block sizes of 2, 4, and 6) with 1:1 allocation between the intervention group and treatment-as-usual group. Because of the nature of the intervention, it was not possible to blind the participant to their treatment allocation, although the research assistants who helped in distributing the questionnaires and the biostatistician who analyzed the data were blinded to patient allocation groups. Moreover, the research assistants who assessed primary and secondary outcomes at baseline and follow-up assessments were blinded to the allocation groups.

### Intervention group

The present study adopted a theory-based intervention based on the HAPA to improve the primary outcome of patients’ medication adherence [33–36]. The intervention comprised a three-session training program based on behavior change techniques (BCTs) that mapped onto key theoretical constructs of the HAPA [35]. Each session lasted approximately 40 min. All sessions were delivered by the first author, who was trained in the delivery of theory-based interventions under the supervision of the last author, an expert in the delivery of health behavior change interventions in clinical settings. Both group and individual sessions were used to improve patients’ attitude and motivation as well as increase medication adherence behavior. The therapist (i.e., the first author) who delivered the training had received prior training in health behavior promotion. Therefore, he used techniques to enhance participants’ engagement, such as motivational interviewing. However, the present study did not request the therapist to utilize any specific techniques, except for BCTs, to help promote participants’ engagement. All sessions allowed for some flexibility to tailor the content to fit participants’ needs, including their health literacy level.

In sessions one and two, outcome expectancies and risk perceptions were targeted. Specifically, in session one, patients’ beliefs regarding their medication taking (e.g., side-effects, withdrawal symptoms, addiction, interactions between medicines) and their RA condition (e.g., risk factors, long-term complications) were elicited. During this session, the facilitator corrected any misunderstanding by providing factual evidence. The facilitator also highlighted the necessity of adherence to medication regimens including addressing underpinning beliefs; for example, concerns about potential adverse effects. The BCTs of information about health consequences (BCT 5.1) and salience of consequences (BCT 5.2) were used in this session. Building on session one, in session two, patients were asked to list the potential benefits of taking their medications and potential risks of not taking their medications. Here, the facilitator encouraged the patients to reflect on the positive and negative consequences of adhering to their medication regimens, and helped address any ambivalence and/or discrepancy in their beliefs. Moreover, and targeting patients’ self-efficacy, role modeling of effective behavior was provided through the use of testimonials, matched by sex and age to the patient, of successful medication adherence-related stories (BCT 16.3). Patients were then encouraged to focus on past successes (BCT 15.3) by either remembering their own successful experiences of taking their medications or by checking some example statements of others’ medication adherence-related success stories. In the final session, session three, planning and self-monitoring were targeted. Here, patients were prompted to make concrete action plans about when, where, and how they would take their medications according to their medication regimen (BCT 1.4). In addition, patients were encouraged to generate their own coping plans (BCT 1.2). For this task, patients were required to write down obstacles that may prevent them from taking their medications according to their regimen and then identify corresponding methods to overcome these obstacles. Finally, a self-monitoring of behavior activity was provided (BCT 2.3). Here, patients were given a medication use calendar with the suggestion to record their medication taking over a 1-month period. Intervention content was not tailored to the individual specifically; however, in the first session participants were able to discuss any issues they were currently having with their condition and medication use and, thus, the facilitator could correct any misunderstanding by providing factual evidence.

### Treatment-as-usual group

Patients in the treatment-as-usual control group received standard care. This included routine education counseling regarding prescribed medications and general information concerning RA delivered by a healthcare professional during usual consultation visits.

### Primary outcome of *change* in medication adherence

#### Medication Adherence Rating Scale

The Medication Adherence Rating Scale (MARS) is a self-report instrument comprising five items assessing the performance of medication adherence. A five-point Likert-type scale was used [37], where a higher score represents better medication adherence. Previous research has shown good psychometric properties for the measure [37]. Participants were instructed to consider their RA medication adherence behaviors when they responded to the MARS. Internal consistency of the scale was satisfactory: *α* = 0.81 in the present study.

### Secondary outcome measures of change in health outcome factors

#### Health Assessment Questionnaire Disability Index

The Health Assessment Questionnaire Disability Index (HAQ) is a self-report instrument, comprising 20 items concerning the functional ability of RA patients [38]. A four-point Likert-type scale was used, where a higher score represents worse functional ability. Previous research has shown good psychometric properties for the measure [38]. Internal consistency of the scale was satisfactory: *α* = 0.89 in the present study.

#### 12-Item Short Form Survey

The 12-Item Short Form Survey (SF-12) is a self-report instrument comprising 24 items. The measure contains a summary assessment of quality of life in two domains: a physical component summary (PCS) and a mental component summary (MCS). The two domains comprise 12 items, which are rated on a variety of response scales, including 2 to 6 categories (i.e., two-point to six-point Likert-type scales). The domain scores are converted into a 0–100 scale, where a higher score indicates better quality of life. Previous research has shown good psychometric properties for the measure [39]. Internal consistency of the two domains was satisfactory: *α* = 0.79 for PCS and 0.84 for MCS in the present study.

#### Visual Analog Scale

The VAS was measured using 1 item that assessed self-reported perceptions of pain. A 0–100-mm scale anchored by “no pain” (score of 0) and “the most extreme pain possible” or “worst imaginable pain” (score of 100) was used [40].

### Secondary outcome measures of theory-based self-regulation factors

The participants were instructed to consider their RA medication adherence behaviors when responding to the following secondary outcome measures.

#### Beliefs about Medications Questionnaire–specific

The Beliefs about Medications Questionnaire-specific (BMQ–specific) is a self-report instrument comprising 10 items. The measure contains two domains of necessity (BMQ-necessity; sample item “My health in the future will depend on my medication”) and concerns (BMQ-concerns; sample item “My medication disrupts my life”). Each BMQ specific domain comprises five items. A five-point Likert-type scale was used, where a higher score on the BMQ-necessity represents more positive beliefs concerning medication and a higher score on the BMQ-concerns represents more negative beliefs [41]. Previous research has shown good psychometric properties for the measure [41, 42]. Internal consistency of the two domains was very good to excellent: *α* = 0.88 for BMQ-necessity and 0.92 for BMQ-concerns in the present study.

#### Intention

Intention to take medication was assessed using five items (sample item “I intend to regularly take medicine in the future”). A five-point Likert-type scale was used, where a higher score represents stronger intention to take medication. Previous research has shown good psychometric properties for the measure [41]. Internal consistency of the scale was excellent: *α* = 0.91 in the present study.

#### Self-efficacy

Self-efficacy was assessed using four items relating to patients’ perceived control and confidence over taking their medication (sample item “For me to take regular medication in the future is difficult/easy”). A five-point Likert-type scale was used, where a higher score represents stronger self-efficacy toward taking medication. Previous research has shown good psychometric properties for the measure [41]. Internal consistency of the scale was very good: *α* = 0.89 in the present study.

#### Action and coping planning

Action and coping planning were assessed using four items for each scale assessing the extent to which patients’ planned to take their medication (action planning; sample item “I have made a detailed plan regarding when to take medication”) and planned to deal with setbacks (coping planning; sample item “I have made a detailed plan regarding what to take medication if I forgot it”). A five-point Likert-type scale was used, where a higher score represents better action planning to take medication. Previous research has shown good psychometric properties for the measure [41]. Internal consistency of the two scales was very good to excellent: *α* = 0.90 for action planning and 0.86 for coping planning in the present study.

#### Self-monitoring

Self-monitoring was assessed using three items related to patients’ self-monitoring concerning the taking of medication (sample item “During the last month, I have consistently monitored when to take medications”). A five-point Likert-type scale was used, where a higher score represents stronger self-monitoring behavior to take medication. Previous research has shown good psychometric properties for the measure [41]. Internal consistency of the scale was very good: *α* = 0.88 in the present study.

#### Self-Report Behavioral Automaticity Index

The Self-Report Behavioral Automaticity Index (SRBAI) is a self-report instrument comprising four items that examine the extent to which a particular behavior (e.g., taking medication) is automatic for an individual [43]. A five-point Likert-type scale was used, where a higher score of the SRBAI represents more automatic action in taking medication. Previous research has shown good psychometric properties for the measure [41]. Internal consistency of the scale was very good: *α* = 0.87 in the present study.

### Control variables

#### Hospital Anxiety and Depression Scale

The Hospital Anxiety and Depression Scale (HADS) is a self-report instrument that contains two domains of anxiety and depression to examine psychological distress. The two domains comprise 14 items which are rated on a four-point Likert-type scale. A higher score of HADS represents a higher level of psychological distress. The psychometric properties of the HADS are satisfactory in its construct validity (i.e., unidimensionality of the anxiety and depression subscale has been supported by the Rasch analyses; the two-factor structure of the HADS has been supported by the confirmatory factor analysis) [44].

#### Background Factors

Body mass index (BMI) was calculated using height and weight (i.e., kg/m^2^). Height and weight of the participants were measured using standard tools by the research associates who had received training in measuring anthropometrics. The number of *disease-modifying anti-rheumatic drugs (DMARDs)* used, *disease duration* (i.e., how long a participant was diagnosed with RA), *age* (in years)**,** *education* (in years), *marital status*, and *sex* (female coded as 0 and male as 1) were collected from the medical records of the patients.

### Sample size calculation

Sample size was calculated according to the primary outcome measure (i.e., the MARS): that is, whether intervention effects on medication adherence can be detected. The sample size calculation was estimated using the following conditions: a medium effect size (*d* = 0.5), power at 90%, and type I error at 0.05 in a two-sided test. It was estimated that 86 patients were needed per group. Utilizing a 15% attrition rate, it was determined that the sample size of 100 per treatment was needed.

### Statistical analysis

Participant characteristics and baseline data are described using means and standard deviations (*SD*) for continuous variables and frequency and/or percentages (%) for categorical variables. To evaluate the magnitude of changes in primary (i.e., MARS) and secondary outcomes over time across the two groups, linear mixed models (PROCMIXED macro) were performed while controlling for age, sex, disease-modifying anti-rheumatic drugs used, body mass index, marital status, years of education, depression, anxiety, and disease duration. Summated scores were applied to all the instruments rather than difference scores for the linear mixed-effects models. Linear mixed models were conducted because they adopt robust algorism to estimate values for those who were lost to follow-up and, therefore, fulfilling the principle of intention to treat [45]. The distributions of all the variables were examined using q-q plot and Kolmogrov-Smirnov test. Except for MARS, all other variables had a normal distribution. Therefore, for the analyses using MARS, a log-transformation score on MARS which was normally distributed was applied. The linear mixed models, which handled the missing values in the present study, were performed using SAS version 9.3 (SAS Institute Inc., Cary, NC, USA). To evaluate the effects of the proposed theory in improving medication adherence, a mediation model was performed using MARS assessed at the 6-month follow-up as the dependent variable, treatment group (i.e., intervention vs. treatment-as-usual) as the independent variables, and the following variables assessed at the 3-month follow-up as mediators: BMQ-necessity, BMQ-concern, intention, self-efficacy, action planning, coping planning, self-monitoring, and SRBAI. Moreover, the appropriateness of model was assessed by likelihood ratio test; the normality and homogeneity of variance were checked using residual analysis. A random intercept model was fitted to the data and the intercept was the only variable that allowed to be varied. In addition, all the numerical variables in the linear mixed-effects models were centered to avoid multicollinearity. The mediation model was performed using IBM SPSS 23.0 (IBM Corp., Armonk, NY) with the PROCESS macro (model 4). A bootstrapping method with 10,000 resamples was used to estimate standard errors of indirect effects. More specifically, bias-corrected bootstrapped samples were used to calculate the 95% confidence intervals. All *p* values were two-sided and were evaluated as statistically significant at the 0.05 level.

## Results

In order to check the fidelity of the intervention, 30% of the audio-recorded transcripts of intervention sessions (*n* = 30) were checked by the last author and an independent researcher in terms of adherence to the study protocol. The fidelity of the intervention was designed by the NIH Behavior Change Consortium (NIH BCC) and included elements of study design, provider training, delivery of treatment, receipt of treatment, and enactment of treatment. The last author and an independent researcher coded all study materials (i.e., audiotapes and transcripts) into component BCTs using a recently adapted taxonomy of BCTs [36]. Interrater reliability for each of these transcripts was calculated and found to have a Cohen’s *κ* of > 0.70 for each transcript, demonstrating good interrater reliability of the coding process.

Table 1 presents participant demographic characteristics at baseline, and Table 2 shows descriptive statistics for all outcome measures, including information of internal consistency, across time in the intervention group and the treatment-as-usual group.

**Table 2:** Descriptive statistics for all outcome measures across time in the intervention (INT) and the treatment-as-usual (TAU) groups

Variable (score range)	Group			Mean (SD)		
	*N* = 100 at baseline	*N* at month 3	*N* at month 6	Baseline	Month 3	Month 6
Medication Adherence Rating Scale (5–25)	TAU	93	88	19.9 (8.0)	19.6 (6.9)	19.7 (6.7)
	INT	91	89	19.6 (5.5)	23.8 (5.9)	24.6 (6.0)
HAQ (0–3)	TAU	92	85	0.6 (0.3)	0.6 (0.5)	0.5 (0.4)
	INT	88	86	0.6 (0.4)	0.3 (0.1)	0.2 (0.2)
Short Form-12: PCS (0–100)	TAU	91	89	50.8 (31.8)	50.3 (32.0)	50.0 (32.3)
	INT	89	87	53.4 (34.1)	58.0 (31.0)	61.2 (29.1)
Short Form-12: MCS (0–100)	TAU	93	89	51.9 (28.3)	52.4 (28.5)	52.7 (28.8)
	INT	87	85	55.5 (28.8)	64.9 (25.6)	66.7 (24.1)
Visual analog scale on pain (0–100)	TAU	94	86	42.0 (32.4)	42.3 (31.9)	43.5 (31.8)
	INT	90	81	40.5 (32.4)	30.8 (29.3)	31.4 (20.4)
BMQ-necessity (5–25)	TAU	89	88	13.9 (4.5)	14.1 (4.5)	14.5 (4.7)
	INT	90	90	13.9 (4.4)	17.1 (4.5)	17.3 (4.9)
BMQ-concerns (5–25)	TAU	91	89	16.4 (3.8)	15.7 (4.4)	15.2 (4.3)
	INT	88	83	16.7 (3.6)	10.5 (3.3)	10.0 (5.1)
Intention (1–5)	TAU	95	85	3.1 (1.9)	3.0 (2.0)	2.9 (1.9)
	INT	91	86	3.1 (1.8)	3.5 (2.0)	3.5 (2.1)
Self-efficacy (1–5)	TAU	97	87	2.3 (1.9)	2.3 (1.0)	2.3 (1.1)
	INT	94	85	2.3 (1.2)	3.6 (1.4)	3.8 (1.4)
Action planning (1–5)	TAU	93	88	2.4 (2.0)	2.2 (1.3)	2.1 (1.2)
	INT	91	90	2.4 (1.3)	4.5 (1.9)	4.8 (2.6)
Self-monitoring (1–5)	TAU	93	90	2.6 (1.1)	2.5 (1.2)	2.5 (1.3)
	INT	96	84	2.7 (1.2)	4.3 (1.9)	4.7 (2.7)
Coping planning (1–5)	TAU	92	82	2.4 (2.0)	2.3 (1.2)	2.4 (1.1)
	INT	94	90	2.4 (1.3)	4.3 (1.9)	4.7 (1.5)
Self-Report Behavioral Automaticity Index (1–5)	TAU	95	91	2.8 (1.1)	2.7 (1.3)	2.7 (1.0)
	INT	93	89	2.8 (1.1)	3.2 (1.1)	3.2 (1.3)

Internal consistency presented using Cronbach’s *α*. *HAQ*, Health Assessment Questionnaire Disability Index score; *PCS*, physical component summary; *MCS*, mental component summary; *BMQ*, Beliefs about Medications Questionnaire–specific

Further analyses using all the participants (i.e., *n* = 200) showed that the improvements in the intervention group were found to be significant in the linear mixed-effects models with the restricted maximum likelihood estimation (see Table 3 and Supplementary Tables S1 and S2). The overall interaction effects between intervention and time were significant for all outcome variables (*p <* 0.001). Therefore, the effects of intervention (intervention group vs. treatment-as-usual group) at different time points (3 months and 6 months vs. baseline) were explored. More specifically, the intervention group reported greater improvements compared with the treatment-as-usual group in MARS score: coefficient = 3.89 (SE = 0.83; *p* < 0.001) at 3-month follow-up; coefficient = 4.50 (SE = 0.80; *p* < 0.001) at 6-month follow-up. The intervention group also reported greater improvements compared with the treatment-as-usual group in all the health outcome measures (Supplementary Tables S1 and S2) and proposed theory-based self-regulation variables (Supplementary Tables S3 to S6).

**Table 3:** Linear mixed-effects models that predicted medication adherence, controlling for age, sex, disease-modifying anti-rheumatic drugs used, body mass index, marital status, years of education, depression, anxiety, and disease duration

Variable	MARS		
	*Β*	95% CI	
Group (Ref: TAU)	0.32	− 1.31,	1.95
Time (Ref: baseline)			
3 months	0.01	− 1.15,	1.17
6 months	0.24	− 0.94,	1.42
Group × time			
INT vs. TAU at 3 months	3.89*	2.26,	5.52
INT vs. TAU at 6 months	4.50*	2.93,	6.07
Age	− 0.02	− 0.06,	0.02
Sex (Ref: female)	0.12	− 1.86,	2.10
DMARDs used	0.12	− 0.06,	0.30
Body mass index	− 0.18	− 0.40,	0.04
Marital status (Ref: single)	0.06	− 0.18,	0.30
Years of education	0.02	− 0.12,	0.16
Depression	− 0.08	− 0.14,	− 0.02
Anxiety	− 0.05	− 0.11,	0.01
Disease duration	0.03	− 0.15,	0.21

*Ref.*, reference group for comparison; *TAU*, treatment as usual group; *INT*, intervention group; *MARS*, Medication Adherence Rating Scale. *p values < 0.001

Furthermore, intervention effects on medication adherence scores were found to be mediated by some of the theory-based self-regulatory variables that guided the study (see Table 4). In Table 4, the total effect (i.e., the sum of direct and indirect effects) of the intervention on MARS was found to be mainly contributed by indirect effects (coefficient = 4.49; 95% CI = 2.91, 6.23). Moreover, the direct effect of intervention on MARS was not significant (coefficient = 0.34; SE = 1.12; *p* = 0.76). After separating the total indirect effect, all the mediators except self-efficacy and action planning showed a significant indirect effect.

**Table 4:** Models of the effect of the intervention on medication adherence behavior with mediators of socio-cognitive variables controlling for age, sex, disease-modifying anti-rheumatic drugs used, body mass index, marital status, years of education, depression, anxiety, and disease duration

	Coefficient	SE	*t*	*p*
Total effect of the intervention on MARS at 6 months	4.83	0.84	5.78	<0.001
Direct effect of intervention on MARS at 6 months	0.34	1.12	0.30	0.76
Indirect effect of intervention on MARS at 6 months	Coefficient	Boot SE	Boot LLCI	Boot ULCI
Total indirect effect of intervention on MARS at 6 months	4.49	0.84	2.91	6.23
Indirect effect of intervention for each individual mediator	Coefficient	Boot SE	Boot LLCI	Boot ULCI
BMQ-necessity at 3 months	0.19	17	0.13	0.57
BMQ-concerns at 3 months	− 0.76	0.38	− 1.60	− 0.10
Intention at 3 months	1.45	0.51	0.55	2.52
Self-efficacy at 3 months	0.49	0.52	− 0.57	1.52
Action planning at 3 months	1.22	0.69	− 0.03	2.66
Coping planning at 3 months	1.18	0.57	0.05	2.74
Self-monitoring at 3 months	1.42	0.56	0.42	2.64
SRBAI at 3 months	1.70	0.44	0.90	2.62

*MARS*, Medication Adherence Rating Scale; *BMQ*, Beliefs about Medications Questionnaire–specific; *SRBAI*, Self-Report Behavioral Automaticity Index; *SE*, standard error

Total effect of the intervention on MARS includes both direct and indirect effects of intervention on MARS. Indirect effect of intervention on MARS includes each mediator’s indirect effect; therefore, a total indirect effect can be computed by summing up all the mediators’ indirect effects

## Discussion

The results of the present study showed that the intervention significantly improved patients’ medication adherence scores. Specifically, RA patients in the intervention group had nearly perfect adherence scores (24.55 out of 25) at 6 months, as well as produced more positive health outcomes and social cognitions. Moreover, the mediation effect of the theory-based self-regulation factors on medication adherence was assessed where it was found that medication beliefs, intention, coping planning, self-monitoring, and behavioral automaticity were significant mediators of the intervention effect on medication adherence scores. A number of studies [13–16] have designed different treatments aimed at improving medication adherence for RA patients. However, studies have rarely examined the process in the theory-proposed mechanisms to explain treatment effects. Thus, the present study extends current understandings of the key determinants in this context.

The current intervention included behavior change strategies that targeted both motivational (e.g., outcome expectancies) and volitional (e.g., planning, self-monitoring) components to improve behavior. This is consistent with other health programs [46–48]. The HAPA makes the distinction between motivational and volitional phases involved in the change process, where constructs like outcome expectancies and risk perceptions help to form an intention in the motivational phase; self-efficacy, planning, and self-monitoring help to enact intentions in the volitional phase; and intention is the bridge between the motivational and volitional phases [26–29]. Based on this, intervention design needs to target the appropriate components within each phase that are most likely to move the individual further toward goal attainment. Given RA patients may not be fully motivated to adhere to their medication regimen, the current intervention first targeted strategies to promote intention formation and then moved to using strategies that focused on helping the patients to enact their intentions. The findings of the mediation effect of the theory-based self-regulation factors on medication adherence supported adopting this approach.

Further, it has been demonstrated that interventions that incorporate more behavior change techniques have larger effects compared with interventions that incorporate fewer techniques [25]. Thus, in the context of this study, it seems that the use of multiple techniques including getting patients to explore their beliefs about taking their medications and getting them to plan and monitor their performance is useful. The latter strategies of planning and monitoring may be especially important given they are shown to help in building habits [26] and, thus, more automatic performance of the behavior, and that findings of the present study showed intervention effects to be mediated by behavioral automaticity.

Given these findings, it is likely that self-regulation factors are important contributors to RA patients’ medication adherence and related health outcomes. The intervention effects found in our study are in line with prior research findings. That is, beliefs about medications are key factors for RA patients to improve their medication adherence [16]. Subsequently, the improved medication adherence may further elevate the health, such as quality of life, for RA patients [7–9].

## Limitations and conclusion

Despite the promising results of the intervention, findings of the present study should be interpreted in light of its limitations. Participants were recruited from Qazvin City; thus, findings may not be representative of those living in more rural areas and to other ethnic groups. Another limitation that should be noted is the use of self-report measures for medication adherence, which may be prone to memory or social desirability bias. As an alternative to self-report, objective instruments could be used, such as medication possession rate, electronic drug monitor, or serum concentration of medication on medication adherence. However, it should be noted that previous research has shown significant associations between MARS and objectively assessed medication adherence [37]. Nevertheless, the lack of objective measures of disease activity and laboratory work in the present study is an important limitation. Further, placebo effects cannot be ruled out given participant blinding was not possible. Moreover, physical and laboratory data (e.g., Disease Activity Score, blood markers of ESR and CRP) were not collected in the present study. Therefore, it is unclear whether intervention effects could affect these physical aspects, especially the disease activity which is a significant confounder in the present study. Following this, a longer period of time for follow-up assessments is suggested to investigate long-term maintenance effects of the intervention.

This theory-based intervention resulted in an improvement in medication adherence behavior and improved health outcomes and social cognitions, and this was evident both in the short and long term. In addition, the present study attempted to further elucidate the mechanisms of changing medication adherence behaviors in a clinical sample of patients at risk for nonadherence behaviors. Overall, the results of the present study support the use of a theory-based intervention for improving medication adherence among RA patients and make a contribution to the cumulative knowledge about self-regulatory processes in health behavior change.

## Electronic supplementary material

ESM 1 (DOCX 75 kb)

## Data Availability

The authors commit to making the relevant anonymized patient level data available on reasonable request.
