# Supplementary material for: A theory-based intervention to promote medication adherence in patients with rheumatoid arthritis: A randomized controlled trial
Source: Clin Rheumatol. 2020 Jun 25;40(1):101–11. doi: 10.1007/s10067-020-05224-y (PMC7782392; doi:10.1007/s10067-020-05224-y)
Supplement: Supplementary file 1 — (DOCX 75 kb) [file 10067_2020_5224_MOESM1_ESM.docx]

**Supplementary Table S1** *Linear mixed effects models that predicted secondary outcomes of health assessment and pain, controlling for age, sex, disease-modifying anti-rheumatic drugs used, body mass index, marital status, years of education, depression, anxiety, and disease duration*

| Variable | HAQ | | | VAS | | |
| --- | --- | --- | --- | --- | --- | --- |
|  | Β | 95% CI | | Β | 95% CI | |
| Group (Ref: TAU) | -0.09 | -0.25, | 0.07 | -0.85 | -2.85, | 1.15 |
| Time (Ref: baseline) |  |  |  |  |  |  |
| 3 months | -0.19* | -0.29, | -0.09 | 0.36 | -2.38, | 3.10 |
| 6 months | -0.01 | -0.09, | 0.07 | 1.55 | -2.39, | 5.49 |
| Group×time |  |  |  |  |  |  |
| INT vs. TAU at 3 months | -0.12 | -0.24, | 0.00 | -11.72* | -18.40, | -5.04 |
| INT vs. TAU at 6 months | -0.33* | -0.43, | -0.23 | -9.66 | -16.36, | -2.96 |
| Age | 0.05 | -0.01, | 0.11 | 0.42 | -0.25, | 1.09 |
| Sex (Ref: female) | -0.15 | -0.37, | 0.07 | -9.28 | -20.31, | 1.75 |
| DMARDs used | 0.03 | -0.01, | 0.07 | -2.38 | -6.14, | 1.38 |
| Body mass index | -0.04 | -0.10, | 0.02 | -3.24 | -7.08, | 0.60 |
| Marital status (Ref: single) | 0.01 | -0.09, | 0.11 | -0.40 | -1.73, | 0.93 |
| Years of education | 0.04 | -0.21, | 0.29 | 0.07 | -0.32, | 0.46 |
| Depression | -0.05 | -0.11, | 0.01 | 0.10 | -0.02, | 0.22 |
| Anxiety | 0.01 | -0.13, | 0.15 | -0.05 | -0.13, | 0.03 |
| Disease duration | 0.08 | -0.14, | 0.30 | 0.02 | -0.08, | 0.12 |

*Note.* Ref. = reference group for comparison; TAU=treatment as usual group; INT= intervention group; HAQ= Health Assessment Questionnaire Disability Index score; VAS= Visual Analogue Scale (VAS) on pain. **p* values < 0.001.

**Supplementary Table S2** *Linear mixed effects models that predicted secondary outcome of quality of life, controlling for age, sex, disease-modifying anti-rheumatic drugs used, body mass index, marital status, years of education, depression, anxiety, and disease duration*

| Variable | PCS | | | MCS | | |
| --- | --- | --- | --- | --- | --- | --- |
|  | Β | 95% CI | | Β | 95% CI | |
| Group (Ref: TAU) | 0.06 | -2.78, | 2.90 | 2.39 | -4.72, | 9.50 |
| Time (Ref: baseline) |  |  |  |  |  |  |
| 3 months | 0.53 | -2.61, | 3.67 | 0.60 | -3.49, | 3.61 |
| 6 months | 0.91 | -1.81, | 3.63 | 0.96 | -2.51, | 4.43 |
| Group×time |  |  |  |  |  |  |
| INT vs. TAU at 3 months | 5.45 | 0.98, | 9.92 | 9.22* | 4.18, | 14.26 |
| INT vs. TAU at 6 months | 6.33 | 2.06, | 10.60 | 8.10 | 2.73, | 13.47 |
| Age | -0.18 | -0.49, | 0.13 | -0.14 | -0.39, | 0.11 |
| Sex (Ref: female) | -2.05 | -6.93, | 2.83 | 3.14 | -4.72, | 11.00 |
| DMARDs used | 2.14 | -1.90, | 6.18 | 1.66 | -1.91, | 5.23 |
| Body mass index | -1.24 | -3.00, | 0.52 | 1.42 | -0.29, | 3.13 |
| Marital status (Ref: single) | 0.04 | 0.00, | 0.08 | 0.13 | -0.44, | 0.70 |
| Years of education | 0.11 | -0.38, | 0.60 | 0.10 | -0.45, | 0.65 |
| Depression | -0.11 | -0.21, | -0.01 | -0.21 | -0.35, | -0.07 |
| Anxiety | -0.03 | -0.07, | 0.01 | -0.05 | -0.09, | -0.01 |
| Disease duration | 0.01 | -0.09, | 0.11 | 0.04 | -0.21, | 0.29 |

*Note.* Ref. = reference group for comparison; TAU=treatment as usual group; INT= intervention group; PCS= Physical Component Summary in the Short Form-12; MCS= Mental Component Summary in the Short Form-12. **p* values < 0.001.

**Supplementary Table S3** *Linear mixed effects models that predicted beliefs about medicines controlling for age, sex, disease-modifying anti-rheumatic drugs used, body mass index, marital status, years of education, depression, anxiety, and disease duration*

| Variable | BMQ-Necessity | | | BMQ-Concerns | | |
| --- | --- | --- | --- | --- | --- | --- |
|  | Β | 95% CI | | Β | 95% CI | |
| Group (Ref: TAU) | 0.19 | -1.10, | 1.48 | -0.06 | -1.22, | 1.10 |
| Time (Ref: baseline) |  |  |  |  |  |  |
| 3 months | 0.22 | -0.62, | 1.06 | -0.82 | -1.78, | 0.14 |
| 6 months | 0.25 | -0.63, | 1.13 | -1.24 | -2.24, | -0.24 |
| Group×time |  |  |  |  |  |  |
| INT vs. TAU at 3 months | 2.97* | 1.74, | 4.20 | -5.10* | -6.45, | -3.75 |
| INT vs. TAU at 6 months | 3.10* | 1.81, | 4.39 | -5.59* | -6.96, | -4.22 |
| Age | 0.08 | -0.04, | 0.20 | 0.02 | -0.02, | 0.06 |
| Sex (Ref: females) | 0.22 | -1.39, | 1.83 | -0.36 | -1.65, | 0.93 |
| DMARDs used | 0.28 | -0.07, | 0.63 | 0.16 | -0.06, | 0.38 |
| Body mass index | 0.04 | -0.14, | 0.22 | -0.02 | -0.16, | 0.12 |
| Marital status (Ref: single) | 0.03 | -0.01, | 0.07 | 0.02 | -0.04, | 0.08 |
| Years of education | 0.03 | -0.01, | 0.07 | -0.04 | -0.08, | 0.00 |
| Depression | 0.02 | -0.02, | 0.06 | 0.03 | 0.01, | 0.05 |
| Anxiety | 0.01 | -0.11, | 0.13 | 0.02 | -0.08, | 0.12 |
| Disease duration | 0.01 | -0.15, | 0.17 | 0.02 | -0.10, | 0.14 |

*Note.* Ref. = reference group for comparison; TAU=treatment as usual group; INT= intervention group; BMQ=Beliefs about Medicines Questionnaire specific. **p* values < 0.001.

**Supplementary Table S4** *Linear mixed effects models that predicted intention and self-efficacy controlling for age, sex, disease-modifying anti-rheumatic drugs used, body mass index, marital status, years of education, depression, anxiety, and disease duration*

| Variable | Intention | | | Self-efficacy | | |
| --- | --- | --- | --- | --- | --- | --- |
|  | Β | 95% CI | | Β | 95% CI | |
| Group (Ref: TAU) | 0.08 | -0.14, | 0.30 | 0.01 | -0.21, | 0.23 |
| Time (Ref: baseline) |  |  |  |  |  |  |
| 3 months | 0.01 | -0.19, | 0.21 | 0.09 | -0.15, | 0.33 |
| 6 months | 0.12 | -0.06, | 0.30 | 0.13 | -0.09, | 0.35 |
| Group×time |  |  |  |  |  |  |
| INT vs. TAU at 3 months | 0.45 | 0.16, | 0.74 | 1.24* | 0.93, | 1.55 |
| INT vs. TAU at 6 months | 0.53* | 0.31, | 0.75 | 1.39* | 1.04, | 1.74 |
| Age | 0.01 | -0.03, | 0.05 | -0.02 | -0.10, | 0.06 |
| Sex (Ref: females) | 0.02 | -0.16, | 0.20 | 0.24 | -0.21, | 0.69 |
| DMARDs used | -0.01 | -0.11, | 0.09 | -0.02 | -0.10, | 0.06 |
| Body mass index | -0.02 | -0.10, | 0.06 | 0.03 | -0.07, | 0.13 |
| Marital status (Ref: single) | -0.01 | -0.07, | 0.05 | 0.02 | -0.10, | 0.14 |
| Years of education | 0.02 | -0.02, | 0.06 | 0.01 | -0.05, | 0.07 |
| Depression | -0.20* | -0.22, | -0.18 | 0.02 | -0.06, | 0.10 |
| Anxiety | -0.03 | -0.05, | -0.01 | -0.05 | -0.09, | -0.01 |
| Disease duration | 0.09 | -0.20, | 0.38 | -0.11 | -0.56, | 0.34 |

*Note.* Ref. = reference group for comparison; TAU=treatment as usual group; INT= intervention group. **p* values < 0.001.

**Supplementary Table S5** *Linear mixed effects models that predicted action and coping planning controlling for age, sex, disease-modifying anti-rheumatic drugs used, body mass index, marital status, years of education, depression, anxiety, and disease duration*

| Variable | Action planning | | | Coping planning | | |
| --- | --- | --- | --- | --- | --- | --- |
|  | Β | 95% CI | | Β | 95% CI | |
| Group (Ref: TAU) | 0.07 | -0.22, | 0.36 | 0.10 | -0.15, | 0.35 |
| Time (Ref: baseline) |  |  |  |  |  |  |
| 3 months | 0.06 | -0.21, | 0.33 | 0.07 | -0.15, | 0.29 |
| 6 months | 0.11 | -0.20, | 0.42 | 0.14 | -0.11, | 0.39 |
| Group×time |  |  |  |  |  |  |
| INT vs. TAU at 3 months | 2.34* | 1.91, | 2.77 | 1.90* | 1.53, | 2.27 |
| INT vs. TAU at 6 months | 2.66* | 2.27, | 3.05 | 2.32* | 1.97, | 2.67 |
| Age | -0.01 | -0.07, | 0.05 | -0.04 | -0.12, | 0.04 |
| Sex (Ref: females) | 0.02 | -0.27, | 0.31 | -0.11 | -0.40, | 0.18 |
| DMARDs used | -0.07 | -0.17, | 0.03 | -0.03 | -0.09, | 0.03 |
| Body mass index | 0.02 | -0.02, | 0.06 | 0.04 | -0.02, | 0.10 |
| Marital status (Ref: single) | 0.01 | -0.07, | 0.09 | 0.04 | -0.08, | 0.16 |
| Years of education | 0.03 | -0.01, | 0.07 | 0.02 | 0.00, | 0.04 |
| Depression | -0.01 | -0.03, | 0.01 | -0.04 | -0.10, | 0.02 |
| Anxiety | -0.03 | -0.07, | 0.01 | -0.04 | -0.18, | 0.10 |
| Disease duration | 0.04 | -0.16, | 0.24 | 0.01 | -0.05, | 0.07 |

*Note.* Ref. = reference group for comparison; TAU=treatment as usual group; INT= intervention group. * *p* values < 0.001.

**Supplementary Table S6** *Linear mixed effects models that predicted self-monitoring and behavioral automaticity controlling for age, sex, disease-modifying anti-rheumatic drugs used, body mass index, marital status, years of education, depression, anxiety, and disease duration*

| Variable | Self-monitoring | | | SRBAI | | |
| --- | --- | --- | --- | --- | --- | --- |
|  | Β | 95% CI | | Β | 95% CI | |
| Group (Ref: TAU) | 0.18 | -0.13, | 0.49 | 0.04 | -0.14, | 0.22 |
| Time (Ref: baseline) |  |  |  |  |  |  |
| 3 months | 0.01 | -0.26, | 0.28 | 0.05 | -0.11, | 0.21 |
| 6 months | 0.06 | -0.14, | 0.26 | 0.12 | -0.02, | 0.26 |
| Group×time |  |  |  |  |  |  |
| INT vs. TAU at 3 months | 1.71 | 1.32, | 2.10 | 0.42 | 0.18, | 0.66 |
| INT vs. TAU at 6 months | 1.99 | 1.64, | 2.34 | 0.50 | 0.28, | 0.72 |
| Age | -0.05 | -0.13, | 0.03 | 0.04 | -0.06, | 0.14 |
| Sex (Ref: females) | -0.09 | -0.40, | 0.22 | 0.09 | -0.13, | 0.31 |
| DMARDs used | 0.03 | -0.03, | 0.09 | 0.01 | -0.07, | 0.09 |
| Body mass index | 0.04 | -0.02, | 0.10 | 0.04 | -0.10, | 0.18 |
| Marital status (Ref: single) | -0.01 | -0.07, | 0.05 | 0.02 | -0.02, | 0.06 |
| Years of education | 0.01 | -0.05, | 0.07 | 0.01 | -0.01, | 0.03 |
| Depression | -0.05 | -0.17, | 0.07 | -0.02 | -0.04, | 0.00 |
| Anxiety | 0.01 | -0.05, | 0.07 | -0.02 | -0.10, | 0.06 |
| Disease duration | 0.16 | -0.41, | 0.73 | 0.04 | -0.14, | 0.22 |

*Note.* Ref. = reference group for comparison; TAU=treatment as usual group; INT= intervention group; SRBAI=Self-report Behavioral Automaticity Index. * *p* values < 0.001.
